# Levels of IL-1β and IL-1 receptor antagonist (IL-1Ra) in Moroccan children admitted to the pediatric emergency department for febrile seizures

**DOI:** 10.1186/s12887-026-06769-8

**Published:** 2026-04-06

**Authors:** Imane Abbari, Sara Missaoui, Halima Kholaiq, Nada Benbouziane, Zahra Aadam, Errami Abderrahmane, Jalila El Bakkouri, Ahmed Aziz Bousfiha, Widad Gueddari

**Affiliations:** 1 Clinical Immunology, Infection and Autoimmunity Laboratory (LICIA), Faculty of Medicine and Pharmacy, Hassan II University, Casablanca, Morocco; 2 Pediatric Emergency Department, Mother and Child Hospital Abdereahim Harouchi Hospital, Ibn Rochd University Hospital, Casablanca, Morocco; 3 Immuno-serology Laboratory of Ibn Rochd University Hospital, Casablanca, Morocco; 4 Pediatric Infectious Diseases and Clinical Immunology Department, Mother and Child Hospital Abdereahim Harouchi Hospital, Ibn Rochd University Hospital, Casablanca, Morocco; 5 Laboratory of Immunopathology-Immunomonitoring-Immunotherapy, Mohammed VI University of Health Sciences (UM6SS), Casablanca, Morocco

**Keywords:** Febrile seizures, Inflammation, Cytokines

## Abstract

**Background:**

Febrile seizures (FS) are events in infancy or childhood, usually between 6 months and 5 years of age, associated with fever but without signs of intracranial infection or a defined seizure cause. Studies suggest that both the inflammatory response and genetic predisposition play crucial roles in FS development. Pro-inflammatory cytokines such as IL-1β and their natural antagonist IL-1Ra are key mediators in this complex interaction.

**Objective:**

This study aimed to evaluate IL-1β, IL-1Ra levels, and the IL-1Ra/IL-1β ratio in Moroccan children with FS and to explore their associations with demographic and clinical parameters.

**Patients and methods:**

This descriptive, cross-sectional study was conducted over six months (September 2022–February 2023) in children aged 6 months to 6 years admitted for FS to the pediatric emergency departments of the University Hospital (CHU) in Casablanca. Serum IL-1β and IL-1Ra levels were measured using enzyme-linked immunosorbent assay (ELISA). Comparative analyses assessed cytokine levels according to age, sex, family history, consanguinity, and temperature at admission.

**Results:**

IL-1β levels were generally low, whereas IL-1Ra levels were markedly elevated, resulting in a high IL-1Ra/IL-1β ratio. IL-1Ra was significantly lower in children from consanguineous families. No significant differences were observed according to other parameter age, sexe, temperature or history of FS or epilepsy.

**Conclusion:**

These findings suggest an imbalance between pro- and anti-inflammatory mediators in FS, reflected by elevated IL-1Ra relative to IL-1β, potentially as a compensatory mechanism limiting IL-1β’s pro-convulsant effects. Genetic and demographic factors may influence this equilibrium, highlighting the need for further studies with larger cohorts to explore underlying mechanisms.

## Introduction

Fever is a systemic response to infection, accompanied by an increased core body temperature of at least 38 °C, triggered by a complex interaction between the immune system and the neuronal circuits. Although it generally confers a survival benefit, the febrile response can occasionally cause adverse events [1].

In young children, fever lowers the seizure threshold, which can lead to the occurrence of febrile seizures (FS) in predisposed children; approximately 2–5% of children between 6 and 60 months of age are reported to experience FS, which occurs during a febrile state and without an apparent central nervous system infection [2].

FS can be simple or complex, based on the clinical state presented by the patient, and is considered benign. However, up to 7% of children with FS develop epilepsy by adolescence [3]. FS occur in both boys and girls, although some studies report a slight predominance in males [2].

The exact pathophysiological mechanisms underlying FS remain incompletely understood. A complex interplay between immune inflammation, cytokine network activation, and genetic factors is involved in the pathogenesis of these conditions [4].

Following infection, both pro-inflammatory and anti-inflammatory cytokines are produced and play a crucial role in regulating the febrile response. Among these pro-inflammatory cytokines, interleukin-1 beta (IL-1β) has been shown to have pro-convulsant effects, whereas its naturally occurring interleukin-1 receptor antagonist (IL-1Ra) has a powerful anticonvulsant action [5]. Various studies suggested that the balance between these cytokines influences inflammatory regulation during febrile episodes and may therefore play a role in the pathogenesis of FS [4].

To our knowledge, no previous study has specifically analyzed the profile of inflammatory cytokines in Moroccan children with febrile seizures, a population underrepresented in previous immunological studies of febrile seizures. Given the central role of the IL-1 axis in the pathophysiology of febrile seizures, and the potential influence of population-specific clinical and familial contexts, this study aimed to investigate plasma levels of IL-1β, IL-1Ra, and the IL-1Ra/IL-1β ratio, and to explore their associations with clinical parameters.

## Patients and methods

### Study design and population

We conducted a descriptive cross-sectional study on children admitted for febrile seizures (FS) to the Pediatric University Hospital (CHU) of Casablanca from September 2022 to February 2023. During this period, 2,328 pediatric emergency admissions were recorded, including 248 cases of seizures occurring in a febrile context.

Among these, 41 children were prospectively included in the present clinical and immunological analysis after application of strict inclusion criteria. Eligible patients were children aged between 6 months and 6 years who fulfilled the international diagnostic criteria for febrile seizures, defined as seizures associated with fever in the absence of central nervous system (CNS) infection, metabolic disorders, or a history of afebrile seizures. Written informed parental consent for serum cytokine measurements was required for inclusion.

Patients with evidence of CNS infection, metabolic disorders, prior afebrile seizures, or who did not meet the inclusion criteria were excluded. Children with known chronic conditions or treatments that could affect immune responses were also excluded.

Lumbar puncture was performed selectively when clinically indicated to exclude central nervous system infection in a limited number of patients. No cerebrospinal fluid cytokine analyses were performed.

### Clinical and demographic data collection

Clinical and demographic data were extracted from patients’ medical records using a standardized data collection form designed for the study. For comparative analyses, patients were divided into subgroups according to relevant clinical and demographic variables: age (< 24 months vs. ≥ 24 months, based on the median value of the cohort), sex (male/female), family history of febrile seizures or epilepsy (yes/no), consanguinity (yes/no), and temperature at admission (normal < 38 °C vs. ≥ 38 °C).

### Cytokines measurement

Blood samples were collected within the first 24 h of seizure onset. Serum was immediately separated by centrifugation and stored at − 80 °C until analysis. Interleukin-1β (IL-1β) and Interleukin-1 Receptor Antagonist (IL-1Ra) levels were quantified using enzyme-linked immunosorbent assay (ELISA) according to the manufacturer’s instructions, with standard curves established for human IL-1β (Abcam, ab108865) and IL-1Ra (Abcam, ab211650).

As no control group was included, cytokine concentrations were interpreted using reference values reported in studies conducted in healthy pediatric populations, as detailed in the Discussion. These reference ranges were used for contextual interpretation only and not as diagnostic thresholds.

### Statistical analysis

Data were analyzed using SPSS software (version 25). Quantitative variables are presented as mean ± standard deviation (SD) or median [interquartile range], as appropriate, and categorical variables as counts and percentages.

Comparative analyses were performed to explore differences in cytokine levels according to clinical and demographic variables (age group, sex, family history of febrile seizures or epilepsy, parental consanguinity, and temperature at admission). Student’s *t*-test was used for normally distributed variables, and the Mann–Whitney *U* test for non-normally distributed variables or small subgroup sizes. Results are reported as *p*-values, with 95% confidence intervals where applicable. No multivariable analyses were performed.

Spearman’s rank correlation coefficients were calculated to assess associations between IL-1β, IL-1Ra, and the IL-1Ra/IL-1β ratio.

Given the descriptive and exploratory design of the study and the marked interindividual variability of cytokine levels, graphical representations were used solely to illustrate data distribution and dispersion. for descriptive purposes only.

### Ethics

The study adhered to the principles of the Declaration of Helsinki and was approved by the Ethics Committee of the Ibn Rochd University Hospital (Approval Date: 2020/ File No 28/20). Written informed consent was obtained from all parents or legal guardians for participation and the disclosure of personal and clinical information.

## Results

### Patient characteristics

The study included 41 children diagnosed with febrile seizures, with a median age of 24 months (range: 6–60 months) and a male predominance (56.1%). Most children experienced simple febrile seizures (65.9%), while 34.1% presented with complex febrile seizures. A family history of febrile seizures was reported in 10 patients (24.4%), and a family history of epilepsy in 8 patients (19.5%). Parental consanguinity was identified in 5 cases (12.2%). Six patients (14.6%) had a history of one or more prior febrile seizures, while 35 (85.4%) were admitted for their first episode.

The mean admission temperature was 38.4 ± 0.9 °C. In a small subset of children, body temperature was below 38 °C at admission, most often due to prior antipyretic administration or spontaneous defervescence before hospital evaluation; in all cases, a febrile episode preceding the seizure was documented based on parental report and medical records. Respiratory infections were the most frequently identified etiology of fever (65.9%). Detailed demographic and clinical characteristics are summarized in Table 1.

**Table 1:** Clinical and demographic characteristics of febrile seizure (FS) children (*N* = 41)

Clinical and demographic characteristics	Number of patients	% or Mean ± SD / Median (Range or IQR)
Age (months)	41	27.2 ± 14.9 ; Median 24 (6–60)
Sex (M/F)	23 / 18	Sex ratio = 1.28
Family history of FS	Yes = 10	24.4%
	No = 31	75.6%
Family history of epilepsy	Yes = 8	19.5%
	No = 33	80.5%
Parental consanguinity	Yes = 5	12.2%
	No = 36	87.8%
Age at onset of FS (months)	41	14 ± 7.7 Median 12 (6–25)
Seizure duration (minutes)	41	11.5 ± 9.2
Seizure type	Simple = 27	65.9%
	Complex = 14	34.1%
Temperature at admission (°C)	41	38.4 ± 0.86
Etiology of fever	Respiratory = 27	65.9%
	Gastro-intestinal = 9	22%
	Ear, nose and throat (ENT) = 4	9.8%
	Urinary = 1	2.4%

Data are presented as mean ± SD, median (range or IQR), or percentages (%), as appropriate

### Inflammatory markers and cytokines

The mean leukocyte count was 13,860 ± 4,120/mm³, and the mean C-reactive protein (CRP) concentration was 56.87 ± 86.32 mg/L, indicating a frequent systemic inflammatory response with wide interindividual variability (Table 2).

**Table 2:** Inflammatory markers and cytokines of febrile seizure (FS) children

Parameter	Number of patients	Mean ± SD or Median (Range)
Leukocytes (/mm³)	41	13,860 ± 4120
CRP (mg/L)	41	56.9 ± 86.3
IL-1β (pg/ml)	41	4.88 ± 8.15
		1.5 (0.48–33.79)
IL-1Ra (pg/ml)	41	1,936.56 ± 2,594.51
		622.7 (221.7–10,697.66)
IL-1Ra / IL-1β ratio	41	1,473.52 ± 2,408.4
		551.4 (8.5–10,004.23)

Data are presented as mean ± SD ; Median (IQR)

*Abbreviations CRP* C-reactive protein, *IL-1β* interleukin-1 beta, *IL-1Ra* interleukin-1 receptor antagonist

Regarding cytokine levels, IL-1β concentrations were generally low, with a median of 1.5 pg/mL (range: 0.48–33.79 pg/mL). Based on reference values reported in healthy pediatric populations, in which IL-1β is typically undetectable or below 3.2 pg/mL, 6 of 41 children had IL-1β levels above this threshold.

In contrast, IL-1Ra concentrations were markedly elevated, with a median of 622.7 pg/mL (range: 221.73–10,697.66 pg/mL). All patients had IL-1Ra levels above reference values reported in healthy children (median ≈ 139 pg/mL).

The IL-1Ra/IL-1β ratio showed substantial interindividual variability, with a median of 551.42 (range: 8.53–10,004.23).

Exploratory correlation analysis did not reveal a significant association between IL-1β and IL-1Ra concentrations (Spearman’s coefficient = − 0.13, *p* = 0.205).

**Fig. 1:**
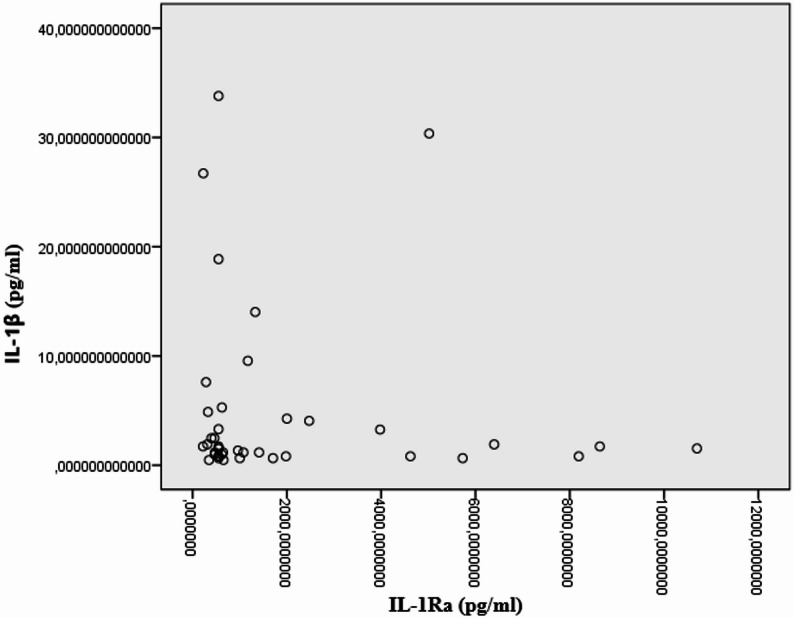
Distribution of IL-1β (pg/ml) and IL-1Ra (pg/ml) concentrations in children with febrile seizures. Scatter plot showing individual serum concentrations of IL-1β and IL-1Ra in the study population (*n* = 41). Each point represents one patient. The figure illustrates the marked interindividual variability of cytokine levels. Visualization is provided for descriptive purposes only

To illustrate the distribution and dispersion of cytokine concentrations, individual values are presented graphically in Fig. 1. These visualizations are provided for descriptive purposes only and reflect the marked biological heterogeneity observed within the cohort.

### Comparison of cytokine levels across clinical and demographic parameters

Comparative analyses of cytokine levels according to age, sex, family history of febrile seizures or epilepsy, consanguinity, and temperature at admission are summarized in Table 3.

**Table 3:** Comparison of cytokine levels across clinical and demographic parameters

Parameter	Subgroups (*n*)	IL-1β (pg/mL, mean ± SD)	*p* (IL-1β)	IL-1Ra (pg/mL, mean ± SD)	*P* (IL-1Ra)	IL-1Ra/IL-1β ratio (mean ± SD)	*p* (ratio)
Age	< 24 months (17)	4.45 ± 7.21	0.508	1732.4 ± 2199.5	0.282	1360.8 ± 1695.4	0.223
	≥ 24 months (24)	5.19 ± 8.90		2081.2 ± 2879.0		1553.4 ± 2841.1	
Sex	Female (18)	3.14 ± 4.49	0.021	2210.5 ± 2737.3	0.504	1556.3 ± 2453.7	0.762
	Male (23)	6.24 ± 10.05		1722.1 ± 2517.9		1408.7 ± 2425.7	
Epilepsy history	Yes (8)	4.96 ± 10.27	0.576	2783.8 ± 3220.3	0.079	1962.2 ± 3413.7	0.183
	No (33)	4.86 ± 7.75		1731.2 ± 2433.7		1355.1 ± 2151.1	
Family history of FS	Yes (10)	4.66 ± 5.39	0.575	1162.2 ± 747.2	0.076	710.4 ± 1086.4	0.252
	No (31)	4.91 ± 8.52		2044.1 ± 2744.4		1579.5 ± 2529.7	
Consanguinity	Yes (5)	3.05 ± 3.72	0.316	681.9 ± 393.1	**0.038**	531.8 ± 612.6	0.111
	No (36)	5.14 ± 8.59		2110.8 ± 2723.9		1604.3 ± 2538.1	
Temperature at admission	Normal (5)	7.14 ± 8.67	0.380	2252.9 ± 3334.6	0.509	2270.3 ± 4332.4	0.042
	Febrile (36)	4.57 ± 8.16		1892.6 ± 2531.0		1362.9 ± 2093.0	

Data are presented as mean ± SD. Comparisons between groups were performed as described in the Methods section. Statistical significance was set at *p*< 0.05

Bold values indicate statistically robust associations, defined by *p* < 0.05 with confidence intervals excluding zero

No statistically significant differences were observed according to age, sex, family history of febrile seizures, or family history of epilepsy, with comparable levels of IL-1β, IL-1Ra, and the IL-1Ra/IL-1β ratio across these subgroups (all *p* > 0.05).

In contrast, parental consanguinity was associated with significantly lower IL-1Ra levels in affected children compared with those from non-consanguineous families (*p* = 0.038). No statistically significant differences were observed for IL-1β or the IL-1Ra/IL-1β ratio according to consanguinity.

Cytokine levels did not differ significantly according to temperature at admission.

Overall, only the association between consanguinity and IL-1Ra reached statistical significance, with confidence intervals excluding zero, whereas other comparisons showed p-values close to the significance threshold but with confidence intervals including zero and were therefore interpreted cautiously.

Subgroup analyses were conducted for exploratory purposes, based on clinical parameters previously implicated in febrile seizure susceptibility, to assess potential variations in immuno-inflammatory profiles.

## Discussion

In this descriptive cohort of Moroccan children with febrile seizures, we observed a cytokine profile characterized by relatively low circulating IL-1β levels, markedly elevated IL-1Ra concentrations, and a high interindividual variability of the IL-1Ra/IL-1β ratio, with a significant association between parental consanguinity and lower IL-1Ra levels.

In our Moroccan pediatric cohort, plasma IL-1β levels were low on average, with considerable interindividual variability. These levels were compared to the reference values reported by for healthy children [5, 6], where IL-1β is typically undetectable or below 3.2 pg/mL in the absence of active inflammation, our results appear only slightly higher overall. This suggests that, although an inflammatory process is present, systemic IL-1β release remains limited or transient in most patients [7–9]. The variability in IL-1β levels observed in our cohort may be due to differences in infection type, age, genetic background or the timing of sample collection. IL-1β peaks rapidly within a few hours of a seizure and declines within 24 h, meaning that samples collected within this timeframe may underestimate peak levels. Technical factors, such as plasma protein binding masking IL-1β detection, may also contribute to low measured concentrations [5].These factors may help to explain discrepancies in previous studies, which have reported heterogeneous findings. For example, several studies in Asia and North Africa, including those from Korea [10], Iran [11] and Egypt [5] (6), have reported significantly elevated levels of interleukin-1 beta (IL-1β) in patients with febrile seizures. However, other studies in these regions, such as those from Iran [12] and Korea [13], as well as in Europe [14, 15], have reported low or non-significant changes.

The activity of IL-1β is counterbalanced by its endogenous antagonist, the IL-1 receptor antagonist (IL-1Ra). IL-1Ra counteracts IL-1β signaling through competitive receptor antagonism, thereby limiting pro-inflammatory and pro-convulsant effects [16].

Unlike IL-1β, IL-1Ra production increases several hours after the onset of inflammation, reflecting a delayed, compensatory anti-inflammatory response [16, 17]. In our study, IL-1Ra levels in plasma were markedly higher than the median reference value reported for healthy children (median ≈ 139 pg/mL) [6]. This increase likely reflects strong activation of the anti-inflammatory response, acting as a compensatory mechanism to counterbalance the neurotoxic and pro-convulsant effects of IL-1β [16](20). Similar findings have been reported in various cohorts, including Asian studies from Korea [13, 18] and European studies from Finland [14, 15]. These findings support the hypothesis of a compensatory anti-inflammatory response follows seizure-induced cytokine release. However, a study conducted in the United States [19] did not observe this increase, potentially due to methodological discrepancies or individual variability. Unlike IL-1β, which typically rises early after a seizure, IL-1Ra peaks later and remains elevated for longer. This could explain the sustained high levels observed in our study [5, 14].

The high IL-1Ra/IL-1β ratio observed in our cohort provides further support for the hypothesis of a strong compensatory anti-inflammatory regulation during febrile seizures. Experimental data indicate that at least a 100-fold excess of IL-1Ra is required to effectively neutralize IL-1β activity [14, 16]. Clinical studies have reported similar or even higher ratios, reaching approximately 250 during initial seizures and up to 665 in recurrent episodes [5, 14]. Consistent with these observations, our findings show a mean IL-1Ra/IL-1β ratio nearly 400-fold higher than IL-1β levels, suggesting an enhanced anti-inflammatory counter-regulatory response to the early pro-inflammatory IL-1β peak [17].

Several clinical studies have suggested that a higher IL-1Ra/IL-1β ratio may be associated with reduced seizure severity or shorter seizure duration, supporting a potential anticonvulsant or protective role of IL-1Ra during febrile seizures [17]. Although the correlation between seizure duration and cytokine levels was not tested in the present study, the elevated IL-1Ra/IL-1β ratio observed in our cohort is consistent with these pathophysiological models and supports the concept of an active compensatory anti-inflammatory response.

In our descriptive exploratory cohort of 41 Moroccan children with febrile seizures, comparative analyses revealed that age, family history of febrile seizures or epilepsy, were not significantly associated with IL‑1β, IL‑1Ra, or their ratio, consistent with previous studies reporting limited effects of these factors on cytokine levels [10, 11, 13, 20]. IL-1β tended to be higher in males (*p* = 0.021). However, the 95% confidence interval included zero, suggesting that this difference should be interpreted cautiously and was therefore not considered statistically robust. This observation may nonetheless suggest a potential sex-related modulation of inflammatory responses, in line with hypotheses of subtle immunogenetic differences [3]. Such variability may partly explain the higher prevalence of febrile seizures among males reported in several populations [11].

The association between parental consanguinity and lower IL-1Ra levels observed in our cohort may reflect underlying genetic factors influencing inflammatory regulation. Consanguinity is known to increase the expression of recessive or homozygous genetic variants, which may affect cytokine production or regulatory pathways, including those involving the IL-1 axis. Polymorphisms in the IL1RN gene have been reported to influence IL-1Ra expression and have been associated with altered inflammatory responses in various populations [5, 21, 22]. Although no genetic analyses were performed in the present study, this finding suggests that familial or genetic background may modulate anti-inflammatory counter-regulation during febrile seizures and warrants further investigation in larger cohorts incorporating genetic approaches.

Regarding temperature at admission, IL‑1β and IL‑1Ra levels were similar between groups, while the IL‑1Ra/IL‑1β ratio tended to be higher in children with “normal” temperature (*p* = 0.042; IC95% including 0), potentially reflecting early compensatory anti-inflammatory activity. These observations underscore the relevance of evaluating cytokine balance rather than absolute concentrations alone [14, 16, 17]. These observations support current pathophysiological models of febrile seizures and underline the importance of considering the balance between pro- and anti-inflammatory mediators rather than isolated cytokine levels.

This study provides the first descriptive characterization of the IL-1β/IL-1Ra axis in Moroccan children with febrile seizures, an underrepresented population in immunological FS research. By jointly assessing IL-1β, IL-1Ra and their ratio, it offers an integrated view of the inflammatory balance and generates hypotheses for future multicenter and genetic studies.

The combined evaluation of IL-1β, IL-1Ra and their ratio provides an integrated view of the balance between pro- and anti-inflammatory responses. The identification of lower IL-1Ra levels in consanguineous families generates novel hypotheses regarding genetic influences on inflammatory regulation.

## Conclusion

Our findings reveal a profile marked by relatively low circulating IL-1β levels and a pronounced elevation of IL-1Ra, resulting in a high IL-1Ra/IL-1β ratio consistent with an active compensatory anti-inflammatory response. The observed association between parental consanguinity and lower IL-1Ra levels suggests that genetic or familial factors may influence inflammatory regulation in febrile seizures. These results generate novel hypotheses and provide a basis for future multicenter, longitudinal, and genetic investigations.

### Limitations

This study has several limitations. The modest sample size limited statistical power for subgroup analyses, and the cross-sectional, single-center design restricts causal inference and generalizability. Cytokine measurements were performed within the first 24 h after seizure onset and were limited to serum samples, which may not fully capture peak IL-1β dynamics or central nervous system inflammation. In addition, the absence of a healthy control group limits direct comparisons; however, published pediatric reference values were used to contextualize the findings. Accordingly, results should be interpreted as descriptive and exploratory.

## Data Availability

The datasets generated and/or analysed during the current study are available from the corresponding author on reasonable request.

## Abbreviations

CRP: C-reactive protein
IL-1β: Interleukin-1 beta
IL-1Ra: Interleukin-1 receptor antagonist
FS: Febrile seizures
ED: Emergency department
CHU: Centre Hospitalier Universitaire
ELISA: Enzyme-linked immunosorbent assay
